# Relationship Between Sleep Quality and Mood: Ecological Momentary Assessment Study

**DOI:** 10.2196/12613

**Published:** 2019-03-27

**Authors:** Sofia Triantafillou, Sohrab Saeb, Emily G Lattie, David C Mohr, Konrad Paul Kording

**Affiliations:** 1 Department of Biomedical Engineering School of Engineering and Applied Sciences University of Pennsylvania Philadelphia, PA United States; 2 Center for Behavioral Intervention Technologies Department of Preventive Medicine Northwestern University Chicago, IL United States; 3 Department of Medical Social Sciences Northwestern University Chicago, IL United States

**Keywords:** sleep, affect, ecological momentary assessment, smartphone, depression, causality

## Abstract

**Background:**

Sleep disturbances play an important role in everyday affect and vice versa. However, the causal day-to-day interaction between sleep and mood has not been thoroughly explored, partly because of the lack of daily assessment data. Mobile phones enable us to collect ecological momentary assessment data on a daily basis in a noninvasive manner.

**Objective:**

This study aimed to investigate the relationship between self-reported daily mood and sleep quality.

**Methods:**

A total of 208 adult participants were recruited to report mood and sleep patterns daily via their mobile phones for 6 consecutive weeks. Participants were recruited in 4 roughly equal groups: depressed and anxious, depressed only, anxious only, and controls. The effect of daily mood on sleep quality and vice versa were assessed using mixed effects models and propensity score matching.

**Results:**

All methods showed a significant effect of sleep quality on mood and vice versa. However, within individuals, the effect of sleep quality on next-day mood was much larger than the effect of previous-day mood on sleep quality. We did not find these effects to be confounded by the participants’ past mood and sleep quality or other variables such as stress, physical activity, and weather conditions.

**Conclusions:**

We found that daily sleep quality and mood are related, with the effect of sleep quality on mood being significantly larger than the reverse. Correcting for participant fixed effects dramatically affected results. Causal analysis suggests that environmental factors included in the study and sleep and mood history do not mediate the relationship.

## Introduction

### Background

Sleep disturbances and poor sleep quality are common and are associated with a wide variety of physical and mental health difficulties [1]. Mood disturbances are often viewed as the hallmark symptoms of mental health disorders such as depression and anxiety. Symptoms of depression and anxiety include disturbances in sleep and poor sleep quality, which are often viewed by patients as being among the more troubling symptoms experienced [2,3]. and are frequently cited as worsening one’s mood. There have been multiple studies demonstrating that sleep disturbances and insomnia relate to the course of depression, including increased prevalence of suicidal ideation and risk of suicide [4-6]. As such, the relationships between sleep and mood have long been examined.

Early investigations of the relationships between mood and sleep typically used retrospective self-report data. For example, Rodin et al [7] conducted 8 interviews over the course of 3 years with participants to determine longitudinal, positive associations between depressed mood and disturbed sleep. As technologies evolved, it became increasingly convenient for researchers to obtain more frequent and accurate assessments of mood and sleep quality. Initially, ecological momentary assessment (EMA) of participants required that participants carry a study-prescribed device such as a personal data assistant. As mobile phones continue to gain popularity, they provide a relatively noninvasive method for collecting data from users. Mobile apps that notify users to complete mood and sleep ratings are often more convenient than paper-and-pencil tracking, and the resulting ratings are more reliable than those in paper-and-pencil charts [8]. Mobile phone data can also provide Global Positioning System (GPS) information on the participants’ daily life that are predictive of their daily mood [9]. Thus, EMA using mobile phones is the state of the art for collecting self-report data.

The increased ease with which we can collect daily assessment data has led to increased focus on the intricate relationships between mood and sleep. Sleep is considered predictive of next-day mood [10-14]. The findings about the effect of mood on sleep are less clear. Some found no significant relations [10], and others found positive affect to be correlated with subsequent sleep quality [12,13]. Most of these studies collected data for 2 weeks or less. Blaxton et al [14] collected data for 6 weeks but did not report results on the relationship between mood and subsequent night sleep. Furthermore, only 1 of these studies included individuals with significant mood symptom variation: Bower et al (2010) compared groups of individuals with major depression and minor depression with healthy controls. Other studies did not report on mood symptoms, whereas Totterdell et al [10] explicitly recruited participants without clinically significant symptoms of depression. A detailed review of the relationship of affect and sleep can be found in the study by Watling et al [15]. Clearly, there have been limitations to past research on the topic as most studies of the relationship of everyday mood and sleep are short and do not differentially assess the mood states of individuals with mood and anxiety disorders. In this work, we attempt to address these shortcomings by using a collection of data over a long period (6 weeks), using mobile phones for reliable data collection, and assessing the differential relationships of sleep and affect in individuals with mood and anxiety disorders as well as in healthy controls.

### Objective

Ultimately, we are asking a causal question. We do not want to know if people with a positive mood sleep better; we want to know if helping them sleep better would improve their mood. The answer to this question can directly inform psychotherapeutic intervention efforts. To ask such causal questions, we can look to statistics for methods that allow correcting for confounders. One popular technique for correcting for confounders is propensity score matching (PSM; [16]). In that approach, we compare days when people are equally likely to engage in a specific behavior (eg, sleep well) based on other measured characteristics. Causal inference from observational data promises to improve the estimation of the causal relationship between sleep and mood.

The aim of this study was to examine the relationship between self-reported sleep quality and self-reported mood in a national sample of adults in the United States, who completed daily ratings via a mobile phone app over 6 weeks. By including roughly equal sizes of depressed, anxious, both depressed and anxious, and control participants, we compared the magnitude of this relationship in these groups. By controlling for a number of possibly confounding factors, we inferred the strength of the *causal* relationship between sleep quality and mood.

## Methods

### Recruitment

Participants were recruited between October 28, 2015, and February 12, 2016, by Focus Pointe Global, a company specializing in participant recruitment [17]. Focus Pointe Global used internet panels of participants as a primary means of recruitment. They sent out emails to these panels with links to the screener questionnaire and made phone calls to potential participants in their in-house registries.

Individuals were eligible for our study if they were aged at least 18 years, able to read and understand English, owned a personal mobile phone with Android 4.4 through 5.1, and had access to Wi-Fi for at least one 3-hour period a day for our data collection software to transfer the data.

To minimize the likelihood of interruption in data collection, we excluded individuals who reported they had been diagnosed with any psychotic disorders or had positive screens for alcohol abuse (Alcohol Use Disorders Identification Test score ≥16; [18]), drug abuse (Drug Abuse Screening Test-10 score ≥6; [19]), suicidal ideation (Patient Health Questionnaire-9 item [PHQ-9] rating ≥1; [20], and Beck Depression Inventory-II item 9 rating ≥2; [21]), or bipolar disorder (Mood Disorder Questionnaire question 1 score ≥7, an endorsement of question 2, and a response of 2 or 3 for question 3 [22]). Potential participants meeting any of these criteria were excluded both for safety reasons, as study staff could not manage problems arising from these conditions in an automated way and to focus the study on depression and anxiety.

Eligible participants consented using procedures approved by the Northwestern University Institutional Review Board, which included descriptions of the data to be gathered along with data security and privacy policies. We selected roughly equal numbers of participants in 4 groups, such that there were wide ranges of depression and anxiety symptoms in the sample. We defined the groups as depressed and anxious (PHQ-9≥10; Generalized Anxiety Disorder-7 item [GAD-7]≥10), depressed only (PHQ-9≥10; GAD-7<10), anxious only (PHQ-9<10; GAD-7≥10), and control (PHQ-9<10; GAD-7<10). These cut-off values for PHQ-9 and GAD-7 were chosen as they have been shown to maximize the sensitivity and specificity for the diagnosis of major depression [20] and GAD (Spritzer et al, 2006), respectively. Table 1 shows the distribution of age, gender, PHQ-9 scores, and GAD-7 scores among groups.

**Table 1:** Distribution of age, gender, Patient Health Questionnaire-9 item (PHQ-9) scores, and Generalized Anxiety Disorder-7 item (GAD-7) scores within the 4 groups of participants.

Group	Age, mean (SD)	Gender (male, female, other), n	PHQ-9^a^, mean (SD)	GAD-7^b^, mean (SD)
Depressed	38.10 (10.30)	6, 43, 2	13.40 (2.90)	5.94 (2.43)
Anxious	36.30 (9.35)	10, 33, 1	7.11 (1.69)	13.36 (2.88)
Depressed and anxious	37.12 (9.56)	8, 44, 0	14.19 (3.45)	14.33 (3.06)
Control	41.42 (11.06)	11, 48, 0	4.47 (3.05)	3.80 (2.95)

^a^PHQ-9: Patient Health Questionnaire-9 item.

^b^GAD-7: Generalized Anxiety Disorder-7 item.

Each participant was enrolled for a period of 6 weeks. Participants were compensated from US $25 up to US $270.40 depending on how long they stayed in the study and how many of the daily questionnaires they answered.

### Data Collection

We collected EMA data to assess the daily mood and sleep quality of the participants. Every day, at 9 am local time, a brief set of questions was launched on the participant’s mobile phone assessing their sleep quality and affect states. A single item prompted participants to rate their sleep quality from 0 to 8 on a 9-point–labeled Likert scale. Mood, stress, energy, and focus levels were also assessed on similar 9-point–labeled Likert scales. The participants also reported the day type (working, day off, half day). In addition to the morning assessments, mood, stress, energy, and focus were assessed 2 more times throughout the day, at 3 pm and 9 pm. Participants could respond immediately to the questions or delay the response until later that day. They were also able to adjust the survey times within a 2-hour window. If they did not answer the questions before midnight, the questionnaire disappeared; on the next day, a new questionnaire was launched asking about the next-day states.

In addition to EMA data, phone sensor data were collected from a range of sensors including GPS, accelerometer, light, microphone, call and short message service events, and Wi-Fi. We used an open-source Android app, *Purple Robot* [23], to collect both sensor and EMA data and to transmit them to our data servers. The data were transmitted via encrypted, password-protected tunnels and then deleted from the phone. The data residing on the server were only accessible to individuals with the proper credentials.

For this study, we included data from sensors known to be related to daily mood and sleep quality. These included physical activity provided by Android’s Activity Recognition application programming interface (API) and the daily weather conditions using the Weather Underground [24].

#### Measured Variables

##### Sleep Quality

Sleep quality was reported on a labeled Likert scale: 0 (extremely poor), 1 (very poor), 2 (poor), 3 (a bit poor), 4 (adequate), 5 (a bit rested), 6 (rested), 7 (very rested), and 8 (extremely rested).

##### Mood, Stress, Energy, and Focus

The 3 self-reported ratings for mood, stress, energy, and focus were averaged for every person and every day. The variables were reported on the following labeled Likert scales:

- Mood: 0 (extremely poor), 1 (very poor), 2 (poor), 3 (a bit poor), 4 (neither), 5 (a bit good), 6 (good), 7 (very good), and 8 (extremely good).
- Stress: 0 (extremely stressed), 1 (very stressed), 2 (stressed), 3 (a bit stressed), 4 (normal), 5 (a bit calm), 6 (calm), 7 (very calm), and 8 (extremely calm).
- Energy: 0 (extremely tired), 1 (very tired), 2 (tired), 3 (a bit tired), 4 (average), 5 (a bit energetic), 6 (energetic), 7 (very energetic), and 8 (extremely energetic).
- Focus: 0 (extremely distracted), 1 (very distracted), 2 (distracted), 3 (a bit distracted), 4 (neither), 5 (a bit focused), 6 (focused), 7 (very focused), and 8 (extremely focused).

The average scores were used in all subsequent analyses. Mood was used in all models to estimate its effect on subsequent sleep quality or to estimate the effect of sleep quality on next-day mood. Stress, energy, and focus were used as possible confounders of mood and subsequent sleep quality and of sleep quality and next-day mood as they are known to be related with both [25].

##### Physical Activity

Physical activity was used as a possible confounder of mood and sleep quality as there is a body of work relating physical activity to well-being [26,27]. The activity of each participant was classified as 1 of the following by the Android’s Activity Recognition API: “in _vehicle,” “on _bicycle,” “on _foot,” “running,” “still,” “tilting,” “walking,” and “unknown.” The sum of “biking,” “running,” and “walking” intervals was used as a measure of physical activity.

##### Weather

Weather is also known to influence affect [28], and is therefore used as a possible confounder. For each participant, the weather report closest to the time the participant visited each location was downloaded from the Weather Underground service. The reports provide a number of attributes, including sky condition and mean temperature. As a measure of weather conditions, we used the mean temperature and the sum of intervals when the sky was clear.

##### Day of the Week and Type of Day

We also included the day of the week (Monday to Sunday) and the type of day (normal [normal work day], partial [partial work day], off [not a work day]) as possible confounders for mood and sleep quality.

### Statistical Analysis

#### Daily Interplay Between Sleep Quality and Mood

Our goal was to examine how sleep quality is related to subsequent mood and also how daily mood affects subsequent sleep quality. To do so, each day’s average mood was paired with (1) sleep quality the following night, to estimate the effect of mood on sleep quality, and (2) sleep quality the previous night, to estimate the effect of sleep quality on mood. All analyses were then performed on both datasets 1 and 2. To estimate the effect of mood on sleep quality, we used dataset 1, with sleep quality as the dependent variable. To estimate the effect of sleep quality on mood, we used dataset 2, with mood as the dependent variable. This allows us to evaluate if the effect of sleep on mood is higher than the effect of mood on sleep. To account for both across-subject and within-subject effects, we used mixed effects models.

#### Within-Subject Normalization

People may respond to self-report scales very differently, in a manner that is consistent for each individual and independent of the specific self-report question [29].

Although mixed effects models use random intercepts to model different baselines, they do not take into account the different ranges used in the personal reports. Therefore, to examine the relationships between the relative personal mood and sleep quality, we normalized the within-person data using the *z*-score of each variable. We then used mixed effects models with varying slopes and fixed (=0) intercepts to calculate the corresponding fixed and random effects. By normalizing within-subject data and using mixed effects models, we tried to quantify the relationship of the *relative personal* mood and sleep quality, defined on the basis of personal variation.

#### Propensity Score Matching

The relationship between sleep quality and mood could be confounded by a number of other factors such as other measures of daily affect (eg, stress), physical activity, environmental factors, and work status. Compared with pairwise models, controlling for these variables bring us closer to the actual *causal* effect of sleep quality on mood and vice versa.

PSM reduces the confounding bias in the estimated effects by controlling for measured confounders and is mainly used for binary treatments (ie, a participant either receives or does not receive treatment). This is done by matching cases (treatment=1) and controls (treatment=0) based on the confounders and then computing the average outcome on both groups. Matching is done based on the propensity score, which is the probability of treatment given the values of the confounders, and can be estimated with a logistic model. This procedure aims to balance the distribution of possible confounds and, thus, reduce the bias of the effect estimate.

Although sleep quality is not a controllable or prescribable treatment for an individual, this analytic method can reduce the confounding bias of the estimated causal effect of sleep quality on mood and vice versa. We applied PSM on each subject. For both models (sleep quality as treatment and mood as outcome; mood as treatment and sleep quality as outcome), the treatment variable within each subject was binarized with respect to the subject mean value. The personal causal effect is estimated as the difference in average outcomes in the 2 matched groups divided by the pooled SD of the 2 matched groups [30]. To check if controlling for confounders changes the estimated causal effects, we compared with the unmatched effect, which is calculated using all the subjects’ data (without matching).

## Results

### Participation

A total of 208 eligible participants were recruited for the study, of which 2 were removed for insufficient data (1 of them did not install the software on their phone and another had invalid GPS data). Of the 206 remaining participants, 170 (82.5%) were females and 36 (17.5%) were males. Their ages ranged between 18 and 66 years, with a mean of 39.3 (SD 10.3). Geographically, they were from 37 different states. Mean depression score (PHQ-9) of the participants was 9.72 (SD 5.06), and mean anxiety score (GAD-7) was 9.0 (SD 5.36).

Participants had a diverse educational background. A total of 4 participants (4/206, 1.9%) had some high school education, 25 (25/206, 12.1%) had completed high school, 73 (73/206, 35.4%) had some college training, 28 (28/206, 13.6%) completed a 2-year college, 49 (40/206, 23.8%) had a Bachelor’s degree, 23 (23/206, 11.2%) had a Master’s degree, and 4 (4/206, 1.9%) had a professional Doctorate degree. Regarding employment status, 126 (126/206, 61.2%) were employed; 43 (43/206, 20.9%) were unemployed; 17 (17/206, 8.3%) had disability, which prevented them from working; and 4 (4/206, 1.9%) were retired. A total of 16 participants (16/206, 7.8%) did not specify their employment status.

The planned duration of the study was 6 weeks, corresponding to 42 sleep quality assessments and 126 mood assessments. However, some participants stopped sending data at various stages of the study, whereas others continued sending data after the completion of 6 weeks, resulting in 6 to 134 report days. The mean reported mood across all participants was 5.0 (SD 1.49), and the mean sleep quality was 4.45 (SD 1.83).

### Daily Interplay Between Sleep and Mood

Using linear mixed effects models [31], we found a significant effect of mood on sleep quality (Table 2, b_1_=.247; *P*<.001) and sleep quality on mood (b_1_=.270; *P*<.001). Although the effect of sleep quality on mood was slightly larger than the opposite effect, this difference was not significant. We emphasize here that the wording *effect* we use here is from the statistical community, and it only corresponds to causality if there are no confounders outside of the participant’s identity.

**Table 2:** Mixed effects model results on mood and sleep quality scores and within-person normalized mood and sleep quality scores.

Predictor	Raw scores		Within-person normalized scores	
	Coefficient (SE)	*P* value	Coefficient (SE)	*P* value
Effect of sleep quality on mood: sleep quality	0.270 (0.013)	<.001	0.344 (0.009)	<.001
Effect of mood on sleep quality: mood	0.247 (0.020)	<.001	0.132 (0.019)	<.001

To see whether our results were affected by participants having different subjective scales for rating mood and sleep quality, we normalized these within each subject and fit mixed effects models to them. The results (Table 2) were different compared with non-normalized data. Importantly, the effect of sleep quality on mood (b_1_=.344; *P*<.001) was now much larger than the effect of mood on sleep quality (b1=.132; *P*<.001). Thus, using normalized scores indicates that the effect of the relative personal sleep quality on mood is on average larger than the opposite. The fact that the precise definition (relative vs absolute) has such a huge influence shows that individual differences in completing questionnaires [29] may lead to spurious correlations between modeled variables.

### Relationship Between Mood and Sleep Quality in the Presence of Confounders

The effect of mood on sleep quality and vice versa can be confounded by other variables that potentially affect both mood and sleep quality, for example, exercise. We used PSM to control for the potential effect of these confounding variables. As possible confounders, we included the following variables: the participant’s mood and sleep quality on the previous day; the participant’s reported stress, energy, and focus levels on the previous day; the participant’s physical activity level on the previous day measured by the phone sensors; weather conditions (mean daily temperature and sky condition); day type (working day, half day, or day off); and the day of the week (calculated based on the EMA data timestamps). Using PSM to control for these variables, we calculated the personal causal effect of mood on sleep quality for each participant, using mood as *treatment* and sleep quality as *outcome,* and the personal causal effect of sleep quality on mood for each participant, using sleep quality as *treatment* and mood as *outcome*. Participants with very low variance in the treatment variable (<0.5) did not admit a reliable treatment discretization and were therefore excluded from the analysis. This setting allows us to correct for confounding bias.

**Figure 1:**
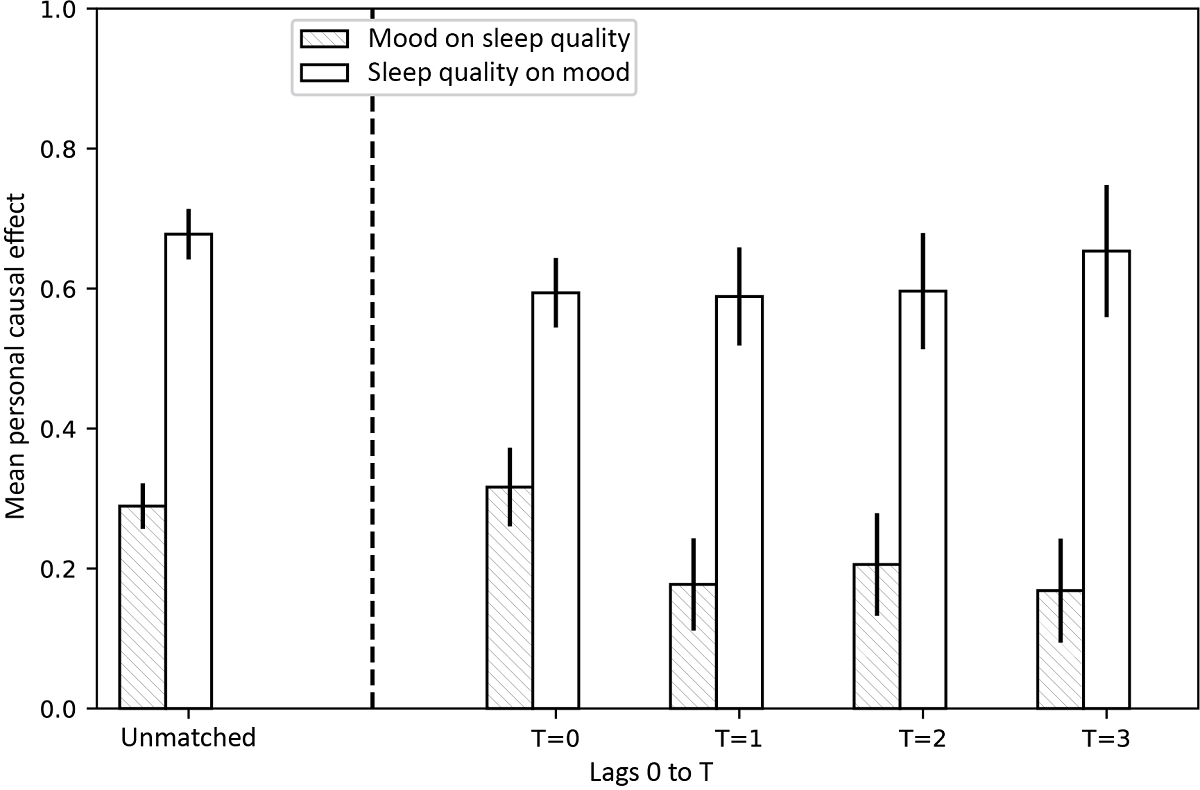
Mean personal causal effect of mood on sleep quality (patterned bar) and sleep quality on mood (empty bar), obtained by 100 bootstraps over participants. Treatment (> mean) and controls (<mean) were matched based on a number of possible confounders, such as weather, stress, focus, energy, day of the week, day type, and activity (T=0, 1, 2, and 3), as well as the mood and sleep quality history (T=1, 2, and 3). Error bars show standard deviations. None of the confounders used seem to confound the effect sizes. Including past sleep quality and mood also does not greatly affect the results. However, the effect of sleep quality on mood was significantly (P<.001) larger than the effect of mood on sleep quality in all cases (unmatched, T =0, 1, 2, 3, 4).

To quantify the effect, we calculated the unmatched scores using all samples from each participant without matching. The effect of sleep on mood quality is significantly (*P*<.001) larger than the effect of mood on sleep quality (Figure 1, left side). This is consistent with the result of the mixed effects models with normalization. For PSM, treatment and control samples for each participant were matched based on the value of confounding variables, and the rest of the samples were discarded. We started by including weather variables, physical activity, stress, focus, energy, day of the week, and day type as confounds. Table 3 describes the confounds used for matching and their correlations with the treatment variable. The results (Figure 1, T=0) did not differ much from the unmatched results. We also included the past values of mood and sleep quality 1 (T=1), 2 (T=2), and 3 (T=3) days before. None of the changes in effect size, as covariates are added, is significant. Therefore, on average, confounds did not affect the relationship between mood and sleep quality in our study.

### Relationship Between Mood and Sleep Quality for Different Groups in Terms of Anxiety and Depression

**Figure 2:**
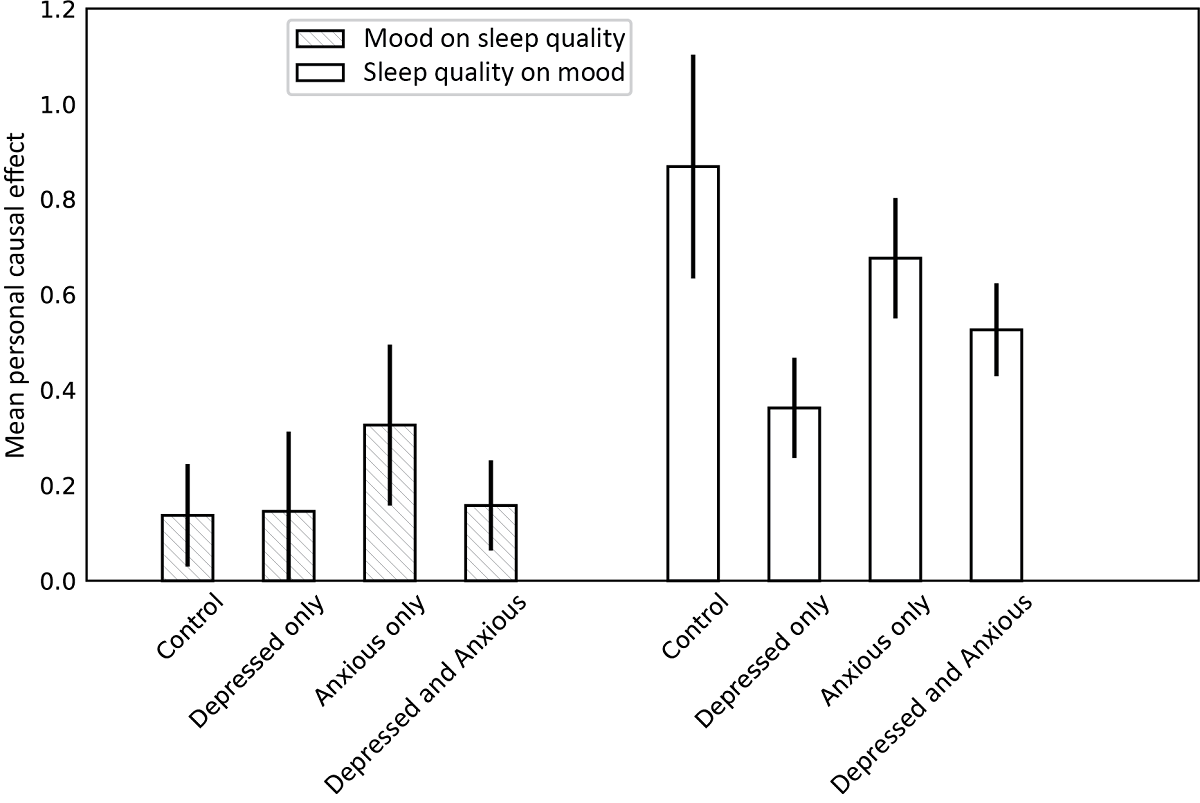
Mean personal causal effect of mood on sleep quality (patterned bars) and sleep quality on mood (empty bars), obtained with propensity score matching by 100 bootstraps over subjects within 4 groups: control, depressed, anxious, and depressed and anxious. Treatment (> mean) and controls (<mean) were matched based on a number of possible confounders, such as weather, stress, focus, energy, day of the week, day type, and activity (T=0, 1, 2, and 3), as well as the mood and sleep quality of the previous day. Error bars show standard deviations. The effect of mood on sleep quality is significantly larger (P<.001) for the anxious group, compared with control. In contrast, the effect of sleep quality on mood is significantly smaller for the depressed (P<.001), anxious (P<.001), and depressed and anxious group (P<.001), compared to control.

To examine whether the causal effect of mood on sleep and vice versa differs for people with different depression and anxiety profiles, we performed the PSM analysis separately in the 4 groups in our study design: *depressed and anxious*, *depressed only*, *anxious only*, and *control*. We matched the subjects based on the confounders shown in Table 3 as well as mood and sleep quality on the previous day (as shown in Figure 1, T=1). We compared the mean estimated causal effect for each of the groups against the control group. For the effect of mood on sleep, the mean personal causal effect is significantly larger for the anxious group compared with control (*P*<.001; Figure 2). However, there was no difference between the control and the rest of the groups. In contrast, the effect of sleep on mood is significantly smaller for depressed, anxious, and depressed and anxious groups (*P*<.001; Figure 2) compared with control. Thus, sleep quality for people with anxiety appears more sensitive to mood fluctuations; on the other hand, sleep quality seems to have a higher effect on the mood of people without anxiety and depression. Moreover, it seems that the relationship of mood and sleep quality is stronger for people with anxiety compared with people with depression.

**Table 3:** Possible confounds and their correlation with treatment, averaged over 206 participants.

Possible confounder	Treatment			
	Sleep quality		Mood	
	Mean (SD)	Number of *P* values (*P*<.05)	Mean (SD)	Number of *P* values (*P*<.05)
Day of the week	0.016 (0.176)	16.0	0.058 (0.170)	20.0
Mean temperature (previous day)	0.017 (0.181)	13.0	−0.019 (0.195)	25.0
Clear sky (previous day)	−0.006 (0.191)	15.0	0.003 (0.192)	17.0
Activity (previous day)	0.013 (0.199)	16.0	0.018 (0.189)	15.0
Day type	0.074 (0.199)	25.0	0.066 (0.199)	20.0
Stress (previous day)	0.160 (0.221)	59.0	0.296 (0.233)	110.0
Energy (previous day)	0.113 (0.228)	44.0	0.220 (0.241)	77.0
Focus (previous day)	0.131 (0.225)	40.0	0.248 (0.227)	80.0

## Discussion

### Principal Findings

We examined the effect of sleep quality on mood and vice versa in a large and diverse sample with a wide range of depression and anxiety symptoms. When we analyzed the data in the full sample, we found these 2 effects to be similar. However, we observed that participants probably used the Likert-type rating scales in different ways. Thus, when individual participant data were normalized and participant data were examined at the individual level, the effect of sleep quality on mood was significantly larger than the effect of mood on sleep quality. This result is consistent with that of previous studies [10-14,32].

We also examined whether the relationship between mood and sleep quality was mediated by other daily variables that can affect both mood and sleep. We examined the roles of daily physical activity, weather conditions, stress, and past mood and sleep quality [26,28,33] and used PSM to obtain a less biased causal effect. We found that the confounders included in the study did not mediate the relationship between sleep quality and mood in either direction. Although we cannot rule out the presence of additional unmeasured confounders, we are confident about these results as this analytic method is known to greatly reduce the bias of the estimated causal effects. These findings suggest that sleep quality has a significant impact on next-day mood, independent of other daily factors, which have valuable clinical implications.

Finally, we examined if the severity of depression or anxiety symptoms affect these relationships. The effect of mood on sleep quality was significantly stronger among anxious participants than among the depressed and control groups. Thus, affect regulation strategies may be effective at improving sleep quality among individuals with elevated symptoms of anxiety [34,35]. However, for the effect of sleep quality on mood, the effect size of the control group was significantly larger than that of the group of individuals with elevated symptoms of anxiety or depression. Overall, these results suggest that the relationship between sleep quality and mood considerably varies depending on the levels of depression or anxiety symptoms.

### Limitations

There are limitations to our study that must be mentioned. First, the study sample is not necessarily entirely representative because of the study design (eg, only Android users) and recruitment procedure (eg, use of a data survey company). However, it is worth noting that relationships between mood and sleep quality did not vary by demographic and socioeconomic factors. In addition, a single item of sleep quality measure was used, and thus, we do not have data available on specific types of sleep disturbances (ie, difficulty falling asleep, staying asleep, early morning wakening, and waking up not feeling rested).

Second, although the PHQ-9 and GAD-7 are validated tools for screening for depressive and anxiety disorders, we did not conduct formal diagnostic interviews. We, therefore, suggest caution in generalizing these findings to clinically diagnosed diagnostic groups.

Finally, we cannot exclude the possibility of additional unmeasured confounders in our analyses, such as social interactions, drug and alcohol consumption, or receiving mental health care. In addition, physical activity was calculated based on phone sensor data, and therefore, it likely overlooked the intervals during which the participants were mobile without carrying their phone. Thus, including additional putative confounders in future studies could help further elucidate the nature of the causal interplay between sleep quality and mood.

### Clinical Implications

Our results indicate that improving sleep quality may have positive outcomes on daily mood. Although the range of sleep quality disturbances is wide, some of them are treatable. Specifically for insomnia, cognitive behavioral therapy for insomnia (CBT-I) has been established as a gold standard treatment [36,37]. Furthermore, past studies have found that the benefits of CBT-I extend beyond sleep to include markers of positive psychological outcomes [38,39] and that these benefits extend to individuals with depression and anxiety [40]. Although CBT-I is unlikely to be prescribed to individuals without diagnosable insomnia, these findings are valuable for elucidating the effect of poor sleep quality on next-day mood. Clinicians should fully assess for sleep disturbances when assessing mood disturbances and are likely to see greater gains in their patients if they address and treat sleep disturbances early in the course of treatment [38,41]. As we found that the effect of sleep quality on mood was significantly greater among control participants compared with participants endorsing elevated levels of depression and/or anxiety, the effect of treating sleep disturbances early in the course of treatment may be particularly relevant for patients endorsing subthreshold mood symptoms.

### Conclusions

Overall, our results suggest that the effect of sleep quality on mood is significantly larger than the effect of mood on sleep quality, even when relevant confounds are considered. However, this relationship can vary across individuals with different levels of depression and anxiety. Moreover, our study highlights the importance of analyzing data at the participant level to account for the individual biases in responding to self-report scales over time. Given the strong effect of sleep quality on next-day mood, clinicians may find enhanced treatment gains by focusing on resolving sleep disturbances early in treatment.

## Abbreviations

API: application programming interface
CBT-I: cognitive behavioral therapy for insomnia
EMA: ecological momentary assessment
GAD-7: Generalized Anxiety Disorder-7 item
GPS: Global Positioning System
PHQ-9: Patient Health Questionnaire-9 item
PSM: propensity score matching
